# Effects of Minority Stress, Group-Level Coping, and Social Support on Mental Health of German Gay Men

**DOI:** 10.1371/journal.pone.0150562

**Published:** 2016-03-04

**Authors:** Frank A. Sattler, Ulrich Wagner, Hanna Christiansen

**Affiliations:** 1 Department of Clinical Child and Adolescent Psychology, Philipps University Marburg, Marburg, Germany; 2 Department of Social Psychology and Center for Conflict Studies, Philipps University Marburg, Marburg, Germany; The University of New South Wales, AUSTRALIA

## Abstract

**Objective:**

According to epidemiological studies, gay men are at a higher risk of mental disorders than heterosexual men. In the current study, the minority stress theory was investigated in German gay men: 1) it was hypothesized that minority stressors would positively predict mental health problems and that 2) group-level coping and social support variables would moderate these predictions negatively.

**Methods:**

Data from 1,188 German self-identified gay men were collected online. The questionnaire included items about socio-demographics, minority stress (victimization, rejection sensitivity, and internalized homonegativity), group-level coping (disclosure of sexual orientation, homopositivity, gay affirmation, gay rights support, and gay rights activism), and social support (gay social support and non-gay social support). A moderated multiple regression was conducted.

**Results:**

Minority stressors positively predicted mental health problems. Group-level coping did not interact with minority stressors, with the exception of disclosure and homopositivity interacting marginally with some minority stressors. Further, only two interactions were found for social support variables and minority stress, one of them marginal. Gay and non-gay social support inversely predicted mental health problems. In addition, disclosure and homopositivity marginally predicted mental health problems.

**Conclusions:**

The findings imply that the minority stress theory should be modified. Disclosure does not have a relevant effect on mental health, while social support variables directly influence mental health of gay men. Group-level coping does not interact with minority stressors relevantly, and only one relevant interaction between social support and minority stress was found. Further longitudinal or experimental replication is needed before transferring the results to mental health interventions and prevention strategies for gay men.

## Objective

In the past two decades, a growing number of studies have been published on mental health disparities between gay men and heterosexual individuals [1–8]. King and colleagues [9] conducted a meta-analysis in which they compared the prevalence of mental disorders in gay and bisexual men with that of heterosexual men. They included North American, European, and Australasian studies. Results indicated that gay men and bisexual individuals have significantly higher 12-month prevalence rates of depression, anxiety disorders, substance dependence, and substance abuse than heterosexual men. Similar results are reported by two other meta-analyses [10–11].

Meyer [10] proposed a model that explains the higher mental morbidity among sexual minority persons compared to heterosexual majority persons. According to Meyer [10], gay men and other sexual minorities are at higher risk of experiencing minority stressors which include victimization [12] (in Meyer’s terminology, prejudice events), rejection sensitivity [13] (in Meyer’s terminology, rejection expectation), concealment of sexual orientation, and internalized homonegativity [14] (in Meyer’s terminology, internalized homophobia). Meyer [10] postulated that these minority stressors have a negative effect on mental health and that coping and social support–at an individual or a group level–moderate this effect negatively. Many studies confirmed that victimization, rejection sensitivity, concealment, and internalized homonegativity predict mental health problems [6, 15–23]. While Szymanski & Owens [24] found that group-level coping in sexual minority women did not moderate the prediction of heterosexist events on distress, no data is yet available on this prediction for gay men. Szymanski & Owens [24] operationalized group-level coping with items based on the sense of community scale for gay men by Proescholdbell, Roosa, and Nemeroff [25]. The dimensions of this original scale are influence (e. g. “How much do you feel your opinion matters to other gay men?”), shared emotional connection (e. g. “In general, how well do you understand other gay men?”), and fulfillment of needs/belonging (e. g. “How much do you feel that you can depend on other gay men?”).

**Fig 1:**
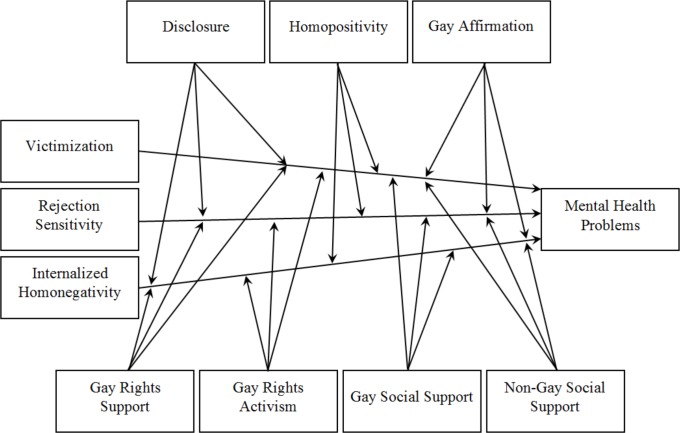
Hypothesized Minority Stress Model.

In the current study a further differentiated minority stress model is proposed (Fig 1). The authors suggest that victimization, rejection sensitivity, and internalized homonegativity are gay-related minority stressors that predict problems in mental health positively. The authors further predict that the effects of minority stressors on mental health problems are lessened by group-level coping and social support. Group-level coping will be operationalized with disclosure, homopositivity, gay affirmation, gay rights support, and gay rights activism. The construct disclosure assesses whether gay men disclosed their sexual orientation to family members, friends, and others [12]. In contrast to Meyer [10], the authors of the current study suppose that disclosing one’s sexual orientation is a group-level coping variable, since disclosure might enable gay men to speak freely about themselves and develop more intimate relationships to friends or partners. Thus, disclosure could prove relevant when exposed to stress. The construct homopositivity captures whether responders agree with positive stereotypes of gay men [26], whereas gay affirmation describes whether they evaluate their gay identity positively and how important it is for them [14]. The current authors suggest that both variables are group-level coping variables, since they enable gay men to develop a positive gay identity which could help to tolerate stress. Gay rights support refers to the responders’ opinion that gay men should have the same rights as heterosexual men [27]. Gay rights activism describes the active behavioral component of gay rights support: it captures whether gay men engage in actions which favor gay rights improvement, such as demonstrating for gay rights [28]. Gay rights support and gay activism are conceptualized as group-level coping variables, since they might enable gay men to change their situation, get a higher social status, and be more tolerant to stress. While Folkman, Lazarus, Gruen, and DeLongis [29] measured coping with variables that assessed “coping and behavioral strategies that people use to manage internal and external demands in a stressful encounter”, it is still unknown whether group-level coping variables are used as a response to stress or if they are unconnected. Nevertheless we suggest that disclosure, homopositivity, gay affirmation, gay rights support, and gay rights activism are connected to the group-level coping variables used by Szymanski and Owens [24] and that they might enable an adaptive coping with stress. Social support is conceptualized as both gay social support and non-gay social support. While gay social support is operationalized as the total number of gay supports, which the individual has, non-gay social support is operationalized as the total number of non-gay supporters (such as women, heterosexual men, or bisexual men), which the individual has. Although satisfaction with heterosexual and sexual minority support was found to be similar among elderly lesbians, gay men, and bisexuals [30], it remains unclear whether there are different effects of gay and non-gay support on the mental health of gay men. Mental health problems are operationalized with the total number of mental health symptoms (such as depression and anxiety symptoms).

In summary, we propose a minority stress model based on Meyer [10] but with some changes. The current minority stress model includes a number of minority stressors, group-level coping variables, social support variables, and mental health. We want to investigate whether minority stressors predict mental health problems and whether group-level coping and social support moderates these predictions.

## Method

The study was approved by the Ethics Committee of the Department of Psychology at the Philipps University Marburg. In the first page of the study participants were informed about the questionnaire (including conditions of participation, anonymity issues, data protection, and duration). They were asked for informed consent by choosing between two online answering options ("Yes, I agree with the conditions" or "No, I do not agree with the conditions"). Only those who provided informed consent were forwarded to the questionnaire. This method of providing online informed consent was approved by the Ethics Committee of the Department of Psychology at the Philipps University Marburg.

### Participants

The study was approved by the Ethics Committee of the Department of Psychology at the affiliated university. Participants were recruited online at the affiliated university, associations of sexual minorities, online news portals, and online gay social networks. The language of the study was German. In the first page of the study participants were informed about the questionnaire (including conditions of participation, anonymity issues, data protection, and duration). In total, 1,933 persons provided informed consent and participated in the study between the 29^th^ of April and the 29^th^ of May 2014. Data of responders in the following conditions were excluded from the analyses: no informed consent (*n* = 30), incomplete questionnaire (*n* = 431), female sex (*n* = 36), non-gay identification (*n* = 131), younger than 18 or older than 77 years (*n* = 7), living in an opposite-sex marriage and a same-sex civil union at the same time (*n* = 1), smoking more than 60 cigarettes per day because of the high improbability (*n* = 5), questionnaire completion in less than five or more than 45 minutes (*n* = 73), and missing values regarding questionnaire completion time (*n* = 31). The final sample consisted of 1,188 self-identified gay men. Their average questionnaire completion time was 14.8 minutes (*SD* = 6.90).

The participants’ mean age was 38 years (*SD* = 11.02; range = 18–73 years). For a detailed display of the sample’s socio-demographic characteristics, see Table 1.

**Table 1:** Socio-Demographic Sample Characteristics.

Characteristic		n	%
Ethnicity			
	Autochthonous German	1,061	89.3
	Immigrant or at least one immigrant parent	127	10.7
Residence			
	Former West Germany	841	70.8
	Former East Germany	254	21.4
	Other country	93	7.8
Number of inhabitants in residing town			
	Less than 1,000	70	5.9
	1,000 to 10,000	136	11.4
	10,000 to 100,000	273	23.0
	100,000 to 1 million	385	32.4
	More than 1 million	324	27.3
Monthly income			
	Less than €500	137	11.5
	€500 to €1,000	189	15.9
	€1,000 to €2,000	323	27.2
	€2,000 to €4,000	418	35.2
	More than €4,000	121	10.2
Education degree			
	No school degree	3	.3
	Junior high school degree	154	13.0
	Middle high school degree	51	4.3
	Senior high school degree	338	28.5
	University degree	531	44.7
	Doctoral degree	111	9.3
Partnership			
	Male partner	608^a^	51.2
	Female partner	8^b^^c^	.7
	No partner	572	48.1
Children			
	At least one child	63	5.3
	No child	1,125	94.7
Religion			
	Protestant	304	25.6
	Roman Catholic	266	22.4
	Muslim	15	1.3
	Orthodox Christian	9	.8
	Buddhist	7	.6
	Jewish	6	.5
	Other religion	27	2.3
	No religion	554	46.6
Religiosity			
	Religious	356	30.0
	Not religious	832	70.0

^a^Of these, n = 143 (23.5%) lived in a civil union. They comprise 12.0% of the total sample.

^b^Of these, n = 5 (62.5%) lived in a marriage. They comprise .4% of the total sample.

^c^Of these, n = 4 were in a partnership with a man and woman.

### Materials and Procedure

The goal in the present study was to create a short and comprehensible questionnaire that would assess a high number of constructs. Therefore, all used inventories were abbreviated and mostly 5-point Likert-type response formats were used if participants were asked for levels of agreement and 4-point scales if they were asked to quantify behaviors.

**Victimization** was assessed with an adapted version of a victimization scale by Herek and Berrill [12]. The original scale consists of 12 items measuring violence and victimization experienced after the age of 16 and a further 12 items assessing victimization in the past year (e. g. “Had verbal insults directed at you?”). The scale has a 3-point response format (0 = *never*, 1 = *once*, 2 = *twice or more*). In the present study, five adapted items that assess victimization since the age of 16 (see S1 Appendix) and an amplified 4-point response scale (1 = *never*, 2 = *once*, 3 = *twice*, 4 = *three times or more*) was used. Herek and Berrill [12] did not report on the psychometric properties of the victimization subscale. The current version of the subscale had a satisfactory internal consistency, with Cronbach’s alpha = .72. A maximum likelihood factor analysis with one extracted factor was applied on the victimization scale. The factor analysis revealed that all items loaded on the same factor (λ = .35 to .84), explaining 42.36% of the variance.

**Rejection sensitivity** was assessed with a modified version of the Gay-Related Rejection Sensitivity Scale [13]. The scale consists of 14 items, each describing a situation that might be interpreted as homonegative (e. g., “A 3-year old [*sic*] child of a distant relative is crawling on your lap. His mom comes to take him away”). The original scale has a 6-point Likert-type format (1 = *very unconcerned* and *very unlikely*, 6 = *very concerned* and *very likely*). In the current study, participants were presented with three slightly modified items (see S1 Appendix) and asked “Do you think this happened because of your sexual orientation?” They were presented with a 5-point Likert-type scale (1 = *strongly disagree*, 5 = *strongly agree*), since we aimed to use comparable item format structures (see above). While the coefficient alpha reported for the original Gay-Related Rejection Sensitivity scale was excellent (.91) [13], the one in the present study reached only .64. This is discussed in the limitations section. A maximum likelihood factorial analysis with one extracted factor was applied on the rejection sensitivity scale. It revealed that all items loaded on the same factor (λ = .49 to .73), explaining 38.58% of the variance.

**Internalized homonegativity** was assessed with an adapted version of the personal homonegativity subscale of the Internalized Homonegativity Inventory (IHNI) [14]. The original IHNI has two more subscales: gay affirmation and morality of homosexuality. It contains 22 items, of which 11 are part of the personal homonegativity subscale (e. g., “I feel ashamed of my homosexuality”). It has a 6-point Likert-type response format (1 = *strongly disagree*, 6 = *strongly agree*). In the current study, a 3-item scale (see S1 Appendix) and a 5-point Likert-type format (1 = *strongly disagree*, 5 = *strongly agree*) was used. The original personal homonegativity subscale has a reported coefficient alpha of .89 [14], and the present one was comparably high, with .86. A maximum likelihood factor analysis with one extracted factor was applied on the internalized homonegativity scale: all items loaded on the same factor (λ = .82 to .84), explaining 68.72% of the variance.

**Disclosure** was assessed with an adapted version of the disclosure scale by Herek and Berrill [12]. The original scale is composed of three items assessing disclosure of one’s sexual orientation (e.g. “If you are gay, lesbian, or bisexual, how much would you say you are “out” to your blood relatives?”). It uses a 10-point Likert-type scale (0 = *not out at all*, 9 = *completely out*). In the present study, the items were slightly adapted (see S1 Appendix) and a 5-point Likert-type answering scale (1 = *not out at all*, 5 = *completely out*) was used. Herek and Berrill [12] did not report on the psychometric properties of their disclosure subscale. The internal consistency of the adapted scale was satisfactory, with an alpha coefficient of .83. A maximum likelihood factorial analysis with one extracted factor was computed for the disclosure scale. Results indicate that all items loaded on the same factor (λ = .75 to .88), explaining 64.61% of the variance.

**Homopositivity** was assessed with a modified version of the Homopositivity Scale (HPS) [26]. The HPS contains nine items that either compare gay men with heterosexual men (e. g., “Gay men tend to be less vulgar than straight men”) or make general statements about gay men without mentioning a comparison group (e. g., “Most gay men have a flawless sense of taste”). The HPS contains a 5-point Likert-type scale (1 = *strongly disagree*, 5 = *strongly agree*). In the present study, three items of the HPS were used, which overtly compared gay men with heterosexual men (see S1 Appendix). The response format was not changed. The coefficient alpha reported for the original HPS was .78 [26], and the one in the current study was comparably high, with .76. A maximum likelihood factorial analysis with one extracted factor was computed for the homopositivity scale. It revealed that all items loaded on the same factor (λ = .64 to .85), explaining 51.95% of the variance.

**Gay affirmation** was assessed with an adapted version of the gay affirmation subscale of the Internalized Homonegativity Inventory [14]. The subscale consists of seven items (e. g., “I am proud to be gay”) and has a 6-point Likert-type response format (1 = *strongly disagree*, 6 = *strongly agree*). In the present study, three items (see S1 Appendix) were used with a 5-point Likert-type scale (1 = *strongly disagree*, 5 = *strongly agree*). The original gay affirmation subscale has a coefficient alpha of .82 [14]; the one in the current study was slightly higher, with .88. A maximum likelihood factor analysis with one extracted factor was conducted for the gay affirmation scale. It revealed that all items loaded on the same factor (λ = .83 to .85), explaining 70.15% of the variance.

**Gay rights support** was assessed with an adapted version of the Support for Lesbian and Gay Human Rights Scale (SLGHR) [31]. The SLGHR contains three factor-analytically derived subscales: social and political rights (e. g., “All employers should strive to develop just and favourable [*sic*] conditions in the workplace for lesbians and gay men”), freedom of expression issues, and privacy of identity. The SLGHR consists of 34 items with a 5-point Likert-type scale (1 = *strongly agree*, 5 = *strongly disagree*). In the current study, three items of the social and political rights subscale were used, which were adapted to address solely support of gay men’s rights (see S1 Appendix). Additionally, the response format was reversed (1 = *strongly disagree*, 5 = *strongly agree*). Ellis and colleagues [31] did not report on the psychometric properties of the SLGHR. The version in the present study had a coefficient alpha of .64. Implications of this low coefficient alpha are discussed in the limitations section as well. A maximum likelihood factorial analysis with one extracted factor was applied on the gay rights support scale. It revealed that all items loaded on the same factor (λ = .51 to .69), explaining 40.06% of the variance.

**Gay rights activism** was assessed with an adapted version of the participation in gay activism scale by Stürmer and Simon [28]. The scale consists of six items that assess if (yes or no) participants have been involved in gay rights activism in the past five years (e. g., “boycott against gay-unfriendly businesses”). If participants indicate “yes”, they are asked for the number of times they have participated in gay rights activism. In the present study, four adapted items were used and a new item was included that assesses gay rights activism in the social media (see S1 Appendix). The item format was modified by using a 4-point scale (1 = *never*, 2 = *once*, 3 = *twice*, 4 = *three times or more*). While the original scale has a coefficient alpha of .65 [28], the adapted version’s was slightly higher, with .68, though this is still not satisfactory. See the limitations section for possible implications of the low coefficient. A maximum likelihood factorial analysis with one extracted factor was conducted on the gay rights activism scale. It revealed that all items loaded on the same factor (λ = .43 to .60), explaining 30.50% of the variance.

**Gay and non-gay social support** were measured with a modified version of the Social Support Questionnaire (SSQ) [27]. The original scale is composed of 27 items (e. g., “Whom can you count on to console you when you are very upset?”) that are answered by listing the initials or relationships of all supporters. A 3-item version was used in the present study. The items were changed to assess the number of perceived supporters, for the sake of anonymity and simplification (see S1 Appendix). Participants inserted a number of participants in a 4-space input field. In the current study, a 3-item subscale assessing the number of gay social supporters was created (see S1 Appendix). In order to detect the number of non-gay supporters, the number of gay supporters was subtracted from the total number of supporters. Subsequently, ranges of both support scales were decreased to a maximum number of 25 supporters, because the previous variation was extensive (participants reported 0 to 9,999 supporters, with an average of 87.3 supporters) and because the median response was 10. The SSQ further assesses satisfaction with support, which was not done by the current authors. The coefficient alpha of the SSQ is .97 [27]. The coefficient alpha of the gay support scale was .77, and the one for the non-gay support scale was .74. A principal component factorial analysis with one extracted factor was computed for the non-gay social support scale: All items loaded on the scale (λ = .61 to .94) and explained 69.92% of the variance. Another principal component factorial analysis with one extracted factor was computed for the non-gay social support scale: All items loaded on the scale (λ = .83 to .97), explaining 88.09% of the variance.

**Mental health** was assessed with adapted items from the Brief Symptom Inventory (BSI) [32, 33] and the Diagnostic Interview of Mental Disorders (DIPS) [34]. The BSI contains 53 items assessing mental problems in the past week (e. g., “loneliness”). Its nine subscales are somatization, obsessive-compulsive, interpersonal sensitivity, depression, anxiety, hostility, phobic anxiety, paranoid ideation, and psychoticism. In the present study, a shortened version of the BSI was used, with three items for each of the nine dimensions. Further, three items related to alcohol dependency were added from the DIPS. They were adapted in order to match the one-week time frame of the BSI. The current mental health scale thus consisted of 30 items in total (see S1 Appendix) and included the BSI’s 5-point Likert-type response format (1 = *not at all*, 5 = *extremely*). In order to validate the mental health scale, based on BSI and DIPS, both an explorative and confirmative factor analysis were computed: in the explorative factor analysis (maximum likelihood) with promax rotation, five factors were extracted. The eigenvalue of the first factor was 12.09, while further eigenvalues dropped considerably to 1.64, 1.41, 1.12, and 1.04. All items were loading positively on the first factor (λ = .40 to .79). In total, 50.04% of the variance for the mental health scale was explained by the five factors. For conformational testing, a maximum likelihood factorial analysis with one factor and an oblique promax rotation (kappa 4) was calculated. 38.29% of the variance in the mental health scale was explained by the factor. All items loaded on the factor (λ = .38 to .80). The authors decided to use the one-factor solution, since eigenvalues of additional factors in the five-factor solution were very low. The global BSI score has a coefficient alpha of .92 [33] and the alcohol dependency scale of the DIPS a Cohen’s kappa coefficient of .70 [34]. The coefficient alpha in the modified scale of mental health problems was highly satisfactory with an alpha of .95.

### Statistical Analyses

A moderated multiple regression was used to test the proposed minority stress model (Fig 1). All analyses were carried out using IBM SPSS Statistics 22.0.

## Results

Bivariate correlations, means, standard deviations, and ranges of the assessed variables are shown in Table 2.

**Table 2:** Correlations of Minority Stress, Group-Level Coping, Social Support, and Mental Health Problems.

Variable	1.	2.	3.	4.	5.	6.	7.	8.	9.	10.	11.
1. Victimization											
2. Rejection sensitivity	.24***										
3. Internalized homonegativity	.07*	.24***									
4. Disclosure	.09**	-.15***	-.48***								
5. Homopositivity	.05	.13***	-.03	.06							
6. Gay affirmation	.06	-.01	-.51***	.34***	.26***						
7. Gay rights support	.11***	.07*	-.16***	.13***	-.002	.23***					
8. Gay rights activism	.27***	.04	-.27***	.38***	.04	.28***	.22***				
9. Gay social support	-.06*	-.17***	-.27***	.30***	.09**	.21***	-.01	.26***			
10. Non-gay social support	-.13***	-.17***	-.16***	.17***	-.09**	.03	.05	-.07*	-.12***		
11. Mental health problems	.33***	.31***	.43***	-.13***	.09**	-.15***	.01	-.02	-.23***	-.24***	
*M*	1.60	2.86	1.68	3.81	2.99	3.15	4.72	2.04	4.75	6.05	1.58
*SD*	.63	.98	.96	1.17	1.05	1.18	.57	.80	5.03	4.77	.59
Range	1–4	1–5	1–5	1–5	1–5	1–5	1–5	1–4	0–25	0–25	1–4.2

Bivariate two-way correlations. M = mean, SD = standard deviation

*p < .05.

**p < .01.

***p < .001.

In the following section, correlations of the minority stressors, group-level coping, social support, and mental health variables are portrayed. Significant two-way correlations which are underneath the threshold of *r* = .10 are interpreted as marginal [35]. Rejection sensitivity was significantly and positively correlated with victimization and internalized homonegativity (*r* = .24, *p* < .001, each), while victimization was marginally associated with internalized homonegativity (*r* = .07, *p* < .05). All three minority stressors (victimization, rejection sensitivity, and internalized homonegativity) were significantly and positively correlated with mental health problems (*r* = .31, *p* < .001 to *r* = .43, *p* < .001). The minority stressors were further significantly and negatively associated with gay and non-gay social support (*r* = -.13, *p* < .001 to *r* = -.27, *p* < .001), with the exception of victimization being only marginally associated with gay social support (*r* = -.06, *p* < .05). Victimization was marginally and positively correlated with disclosure (*r* = .09, *p* < .01). Disclosure was negatively related to rejection sensitivity and internalized homonegativity (*r* = -.15, *p* < .001 and *r* = -.48, *p* < .001, respectively), whereas disclosure was marginally and positively correlated with victimization (*r* = .09, *p* < .01). Notably, homopositivity was significantly and positively correlated with rejection sensitivity (*r* = .13, *p* < .001) and marginally with mental health problems (*r* = .09, *p* < .01). This is inconsistent with the hypothesis that homopositivity is a positive coping variable. Victimization was significantly and positively correlated with gay rights support and gay rights activism (*r* = .11, *p* < .001 and *r* = .27, *p* < .001, respectively). Rejection sensitivity was positively and marginally correlated with gay rights support (*r* = .07, *p* < .05). Internalized homonegativity was significantly and negatively associated with gay affirmation, gay rights support, and gay rights activism (*r* = -.16, *p* < .001 to *r* = -.51, *p* < .001). Most group-level coping variables were associated with one another (*r* = .13, *p* < .001 to *r* = .38, *p* < .001). Only homopositivity was unrelated with disclosure, gay rights support, and gay rights activism (-.002, *p* > .05 to *r* = .06, *p* > .05). Gay social support and non-gay social support were negatively associated with one another (*r* = -.12, *p* < .001). Mental health problems were significantly and negatively correlated with disclosure, gay affirmation, gay support, and non-gay support (*r* = -.13, *p* < .001 to *r* = -.24, *p* < .001). Notably, mental health problems were not significantly associated with gay rights support or gay rights activism (*r* = .01, *p* > .05 and *r* = -.02, *p* > .05, respectively).

A moderated multiple regression was conducted to examine the hypothesized model. Minority stressors, coping, social support, and interactions of the minority stressors with the coping and social support variables were included. Results are indicated in Table 3.

**Table 3:** Moderated Multiple Regression Analysis Predicting Mental Health Problems from Minority Stress, Group-Level Coping, and Social Support.

Predictor	Source of mental health problems
	β
Victimization	.21***
Rejection sensitivity	.12***
Internalized homonegativity	.39***
Disclosure	.09**
Homopositivity	.07**
Gay affirmation	.01
Gay rights support	.04
Gay rights activism	-.001
Gay social support	-.16***
Non-gay social support	-.16***
Interactions^a^	
Total adjusted *R*^2^	.36***

^a^Significant interactions include victimization*homopositivity (β = .08**), rejection sensitivity*gay social support (β = -.10***), internalized homonegativity*disclosure (β = .09*), internalized homonegativity*non-gay social support (β = -.06*). Marginally significant interactions include victimization*non-gay social support (β = -.05^†^) and internalized homonegativity*homopositivity (β = .05^†^).

^†^p < .10

*p < .05.

**p < .01.

***p < .001.

The moderated multiple regression model predicted 36% (*p* < .001) of the variance of mental health problems. Predictions with beta weights lower than .10 are interpreted as marginal predictions [36].

**Fig 2:**
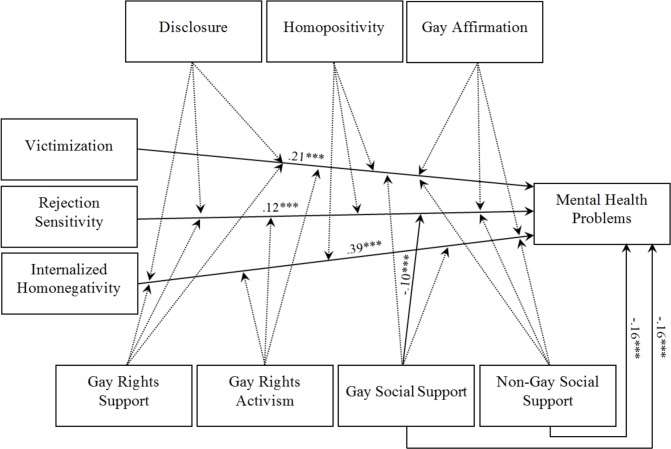
Empirical Minority Stress Model. Only significant predictions and moderations are displayed with beta weights > .10. *p < .05. **p < .01. ***p < .001.

Victimization (β = .21, *p* < .001), rejection sensitivity (β = .12, *p* < .001), and internalized homonegativity (β = .39, *p* < .001) significantly and positively predicted mental health problems (Fig 2). Gay social support (β = -.17, *p* < .001) and non-gay social support (β = -.16, *p* < .001) negatively predicted mental health problems, whereas disclosure and homopositivity marginally predicted mental health problems (β = .09, *p* < .01 and β = .07, *p* < .01, respectively). Gay affirmation (β = .01, *p* > .05), gay rights support (β = .04, *p* > .05), and gay rights activism (β = -.003, *p* > .05) did not predict mental health outcomes. Only one significant moderating relationship with β > .10 was found for rejection sensitivity*gay social support (β = -.10, p < .001) on mental health problems. Marginal positive moderations were found in the case of victimization*homopositivity and internalized homonegativity*disclosure (β = .08, *p* < .01 and β = .09, p < .05, respectively), while a marginal negative moderation was found for internalized homonegativity*non-gay social support (β = -.06, p < .05). The remaining 17 interactions (e. g., victimization*disclosure and rejection sensitivity*gay activism) were not significant.

## Discussion

The current study examined a minority stress model for gay men based on Meyer [10]. This investigation is the first to examine the predictive values of a broad number of minority stressors, group-level coping variables, and social support variables on mental health in German gay men. Results support the hypotheses that the minority stressors victimization, rejection sensitivity, and internalized homonegativity positively predict mental health problems. These findings are consistent with previous minority stress research [6, 15–17, 19–21, 23].

In addition, it was hypothesized that the effect of minority stress on mental health problems is moderated by group-level coping and social support. In accordance with these hypotheses, gay social support moderated the effect of rejection sensitivity on mental health problems. Also, two marginal and positive interactions between victimization*homopositivity and internalized homonegativity*disclosure, as well as a marginal and inverse interaction of internalized homonegativity*non-gay social support were found. These three interactions had low beta weights (β < .10) and are thus interpreted as marginally and being of little relevance [36]. No other significant interactions of minority stressors with group-level coping or social support variables were observed. Since we are the first to examine this complex model of predictions and moderating influences on the mental health of gay men, our findings are in need of future replication. Future research should search for adaptive group-level coping variables including investigations on gay rights support and gay rights activism, since our findings related to those measures are hard to interpret due to their low alpha coefficients.

Another hypothesis was that group-level coping and social support would not predict mental health problems. Indeed, gay affirmation, gay rights support, and gay rights activism did not predict mental health problems. In contrast, disclosure and homopositivity marginally predicted a higher degree of mental health problems, while both gay and non-gay social support predicted a lower degree of mental health problems. Previous studies have failed to find a positive relationship between disclosure and mental health problems [18, 22]. An explanation for the discrepancy might be that in contrast to us, they did not control for possible confounding variables such as internalized homonegativity and that the relationships are very small. In another study, internalized homophobia and emotional support mediated the association between concealment and mental health in a sample of behaviorally bisexual men [37].

We argue that future research on mental health should take into account the predictors victimization, rejection sensitivity, internalized homonegativity, gay social support, and non-gay social support, when comparing the mental health of groups with different sexual orientations (e. g. heterosexuals versus gay men). Also, replicative studies are needed which investigate the influence of disclosure and homopositivity on the mental health of gay men, while controlling for other relevant minority stressors. In order to scrutinize the possible causality of the results, longitudinal studies and ethically approved manipulations of stressors and social support of gay men are needed. If these studies will find similar results, gay men should be supported at diminishing minority stress and at constructively handling it. Also, gay men should be encouraged to broaden their (gay or non-gay) support network. If our moderations are replicated, rejection-sensitive gay men should be especially encouraged to increase their number of gay male supporters.

### Limitations

As with any empirical research, the current study has some limitations. First, due to the online sampling approach, the extent to which the findings can be generalized to the German or Western gay male population is unclear. Second, no causality can be concluded from the current study because of the cross-sectional design. It is thus also possible that mental health problems produce a higher perception of minority stressors. This is likely in the case of depression, since this mental disorder is associated with biases in attention, processing, and memory [38]. Third, although factorial analyses were applied and reliabilities were estimated of all scales, no further validation was done. As a result, the rejection sensitivity, gay rights support, and gay rights activism scales had rather low alpha coefficients [39], meaning that their results cannot be interpreted well. Fourth, gay social support was dependent on non-gay social support because non-gay support was derived from subtracting gay support from total support. Therefore, these scales can only be interpreted with caution. Fifth, the study did not include constructs of resilience. Confusions of the variables in the study (e. g. group-level coping) with resilience are thus possible.

## Conclusion

The findings imply that the minority stress theory [10] should be modified. In accordance with Meyer [10], victimization, rejection sensitivity, and internalized homonegativity predict mental health problems. However, disclosure has a marginal and positive effect on mental health, while social support variables directly influence mental health of gay men. Also, homopositivity predicts mental health problems marginally. Group-level coping and social support do not interact with minority stressors with one exception: gay social support interacts with rejection sensitivity. Further longitudinal or experimental replication is needed before transferring the results to mental health interventions and prevention strategies for gay men.

## Supporting Information

S1 Appendix (DOCX)
