# Supplementary material for: Effects of Minority Stress, Group-Level Coping, and Social Support on Mental Health of German Gay Men
Source: PLoS One. 2016 Mar 4;11(3):e0150562. doi: 10.1371/journal.pone.0150562 (PMC4778907; doi:10.1371/journal.pone.0150562)
Supplement: S1 Appendix — (DOCX) [file pone.0150562.s001.docx]

Appendix

*Items from Minority Stress, Coping, and Social Support Scales*

| Scale | Item |
| --- | --- |
| Victimization | Verbal insults or threatened with physical violence^1^ |
|  | Personal property damaged or destroyed |
|  | Punched, kicked, beaten, or physically wounded in another way^1^ |
|  | Chased or followed |
|  | Sexually harassed or sexually assaulted^1^ |
| Rejection sensitivity | You go get an STD check-up, and the man taking your sexual history is rude towards you. |
|  | You go to a party, and you and your partner are the only gay people there. No one talks to you. |
|  | You are in a locker room in a gym. One guy nearby moves to another area to change clothes.^2^ |
| Internalized homonegativity | I feel ashamed of my homosexuality. |
|  | I sometimes resent my sexual orientation. |
|  | When I think of my homosexuality, I feel depressed. |
| Disclosure | How much are you out to your friends and acquaintances?^2^ |
|  | How much are you out to your relatives?^2^ |
|  | How much are you out to your supervisor, colleagues, and the people with whom you work?^2^ |
| Homopositivity | Gay men are more in touch with their emotions than are straight men. |
|  | Gay men are more articulate than straight men. |
|  | Gay men are better dancers than straight men. |
| Gay affirmation | I am thankful for my sexual orientation. |
|  | I see my homosexuality as a gift. |
|  | I am proud to be gay. |
| Gay rights support | Gay couples should have all the same parenting rights as heterosexuals do (e. g., adoption and fostering).^2^ |
|  | It should be acceptable for gay male couples openly to express their affection for their partners in public (e. g., kissing, holding hands, or embracing each other).^2^ |
|  | All employers should strive to develop just and favorable conditions in the workplace for gay men.^2^ |
| Gay rights activism | Public marches and rallies^1^ |
|  | Information stands or distribution of flyers^2^ |
|  | Collecting signatures (e.g., for a petition) or signing^1^ |
|  | Posting gay rights comments on the Internet (e.g., via Twitter or Facebook)^3^ |
|  | Boycott against [*sic*] gay-unfriendly businesses. |
| Non-gay support | Whom can you really count on to be dependable when you need help (e. g., help with moving or walking your dog)?^2^ |
|  | With how many people can you totally be yourself?^2^ |
|  | On how many people can you count to console you when you are very upset?^2^ |
| Gay support | How many of these people are gay men?^34^ |
| Somatization | Dizziness or nausea^1^ |
|  | Pains in heart or chest |
|  | Hot or cold spells |
| Obsessive-compulsive | Having to check and double-check what you do |
|  | Difficulty making decisions |
|  | Trouble concentrating or remembering things^1^ |
| Interpersonal sensitivity | Strong inhibition when interacting with others |
|  | Feeling emotionally vulnerable |
|  | Feeling inferior to others |
| Depression | Feeling lonely |
|  | Feeling no interest in things |
|  | Feelings of worthlessness |
| Anxiety | Feeling fearful |
|  | Spells of terror or panic |
|  | Feeling so restless you couldn’t sit still |
| Hostility | Feeling easily annoyed or irritated |
|  | Temper outbursts that you could not control |
|  | Getting into frequent arguments |
| Phobic anxiety | Feeling afraid in open spaces |
|  | Having to avoid certain things, places, or activities because they frighten you |
|  | Feeling uneasy in crowds |
| Paranoid ideation | Feeling others are to blame for most of your troubles |
|  | Feeling that that you are watched and talked about by others |
|  | Others not giving you proper credit for your achievements |
| Psychoticism | The idea that someone else can control your thoughts |
|  | The idea that you should be punished for your sins |
|  | The idea that something is wrong with your mind |
| Alcohol dependency | Desire to avoid or reduce negative feelings by consuming alcohol^2^ |
|  | Desire to reduce inhibitions by drinking alcohol^2^ |
|  | Difficulties at handling everyday duties (e. g., work, school, or family) as a result of consuming alcohol^2^ |

*Note.* ^1^The item was formed by combining previously distinct items of the same scale. ^2^The original item was changed. ^3^Newly created item. ^4^The item was presented once after each item of the previous scale.
